# Mitochondria‐Modulating Liposomes Reverse Radio‐Resistance for Colorectal Cancer

**DOI:** 10.1002/advs.202400845

**Published:** 2024-03-23

**Authors:** Junmei Li, Yuhong Wang, Wenhao Shen, Ziyu Zhang, Zhiyue Su, Xia Guo, Pei Pei, Lin Hu, Teng Liu, Kai Yang, Lingchuan Guo

**Affiliations:** ^1^ Department of Pathology the First Affiliated Hospital of Soochow University Soochow University Suzhou Jiangsu 215123 China; ^2^ Department of Oncology Taizhou People's Hospital Affiliated to Nanjing Medical University Taizhou 225300 China; ^3^ State Key Laboratory of Radiation Medicine and Protection School of Radiation Medicine and Protection & School for Radiological and Interdisciplinary Sciences (RAD‐X) Collaborative Innovation Center of Radiation Medicine of Jiangsu Higher Education Institutions Suzhou Medical College Soochow University Suzhou Jiangsu 215123 China

**Keywords:** fractionated radiotherapy, iMOMP, liposome, radio‐resistance, T cell exhaustion

## Abstract

Complete remission of colorectal cancer (CRC) is still unachievable in the majority of patients by common fractionated radiotherapy, leaving risks of tumor metastasis and recurrence. Herein, clinical CRC samples demonstrated a difference in the phosphorylation of translation initiation factor eIF2α (p‐eIF2α) and the activating transcription factor 4 (ATF4), whose increased expression by initial X‐ray irradiation led to the resistance to subsequent radiotherapy. The underlying mechanism is studied in radio‐resistant CT26 cells, revealing that the incomplete mitochondrial outer membrane permeabilization (iMOMP) triggered by X‐ray irradiation is key for the elevated expression of p‐eIF2α and ATF4, and therefore radio‐resistance. This finding guided to discover that metformin and 2‐DG are synergistic in reversing radio resistance by inhibiting p‐eIF2α and ATF4. Liposomes loaded with metformin and 2‐DG (M/D‐Lipo) are thus prepared for enhancing fractionated radiotherapy of CRC, which achieved satisfactory therapeutic efficacy in both local and metastatic CRC tumors by reversing radio‐resistance and preventing T lymphocyte exhaustion.

## Graphical abstract

This work addresses the problem of radio‐resistance caused by activation of intrinsic mitochondrial outer membrane permeability after a single radiotherapy session, then designs and validates the efficacy of PEGylated liposomal drug formulations targeting this mechanism, providing a viable strategy to address the poor efficacy of fractionated radiotherapy in the clinic.

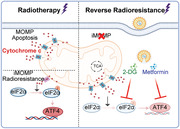

## 1 Introduction

Colorectal cancer (CRC) is one of the most common and deadly malignancies in humans, with incidence rising every year.^[^ ^1^, ^2^ ^]^ Endoscopic resection and extensive surgical excision as the first‐line treatments could effectively inhibit superficial CRC in the early stage.^[^ ^3^, ^4^ ^]^ However, due to the lack of early screening diagnosis, CRC with a rapid proliferation nature is always detected at an advanced stage, which is either not amenable to surgery or results in a postoperative recurrence rate as high as 30–50%.^[^ ^5^, ^6^ ^]^ Radiotherapy could downsize CRC to be prepared for surgery and meanwhile preserve the circumvent sphincter muscle.^[^ ^7^ ^]^ Moreover, clinical evidence has suggested that the addition of radiotherapy, either before or after surgery, could enhance the local control of CRC and patients' prognosis.^[^ ^8^ ^]^ However, the prognosis and overall survival (OS) rate of CRC patients have not been substantially improved.^[^ ^9^ ^]^ The hindrance for the radical elimination of cancer cells as well as the inducement for tumor relapse and metastasis are still enigmas to be clarified to advance the clinical management of CRC.

Studies have demonstrated that the cancer cells that survived treatment would establish therapy resistance and even elevate invasiveness.^[^ ^10^, ^11^ ^]^ Considering the oxygenation efficiency and side effects, clinical radiotherapy is commonly conducted by multiple X‐ray irradiations at a determined dosage, i.e., fractionated radiotherapy.^[^ ^12^, ^13^ ^]^ In this circumstance, the initial X‐ray irradiation may lead to resistance of cancer cells to the subsequent radiotherapy and thus challenge the satisfactory therapeutic outcome and long‐term survival of cancer patients.^[^ ^14^, ^15^ ^]^ Therefore, to fulfill the thorough elimination of CRC by radiotherapy, it is pivotal to study the mechanism underlying the radio‐resistance of cancer cells developed during fractionated radiotherapy and accordingly address radio‐resistance.

Mitochondria not only dominate systemic energy metabolism in live cells but also orchestrate cellular apoptosis and necrosis, which play a crucial role in tumorigenesis.^[^ ^16^, ^17^ ^]^ Conventionally, external stimuli augmenting mitochondrial outer membrane permeability (MOMP) would trigger the release of pro‐apoptotic substances such as cytochrome c (Cyt c) to the cytoplasm, leading to the apoptosis of cancer cells.^[^ ^18^, ^19^, ^20^ ^]^ One of these treatments involves perturbing the homeostasis of calcium ions within and outside mitochondria.^[^ ^21^, ^22^ ^]^ However, recent research discovered that MOMP is adaptively controlled by the Bcl‐2 family effector protein on mitochondria.^[^ ^23^ ^]^ Incomplete MOMP (iMOMP) and insufficient release of Cyt c would induce integrated stress response (ISR), leading to the phosphorylation of the translation initiation factor eIF2α (p‐eIF2α) and overexpression of activating transcription factor 4 (ATF4).^[^ ^23^ ^]^ This adaptive response commits cancer cells to bypass apoptosis, and consequently therapy resistance and cancer invasion. These results suggested that the modulation of MOMP would circumvent radioresistance and achieve potent radiotherapy of cancer.

Metabolic reprogramming has been well‐recognized as a hallmark of cancer cells and the preference for aerobic glycolysis has been recently developed as a therapy target.^[^ ^24^, ^25^ ^]^ Meanwhile, the oxidative respiratory chain has also been aimed to disrupt the mitochondrial function of cancer cells.^[^ ^26^ ^]^ However, to the best of our knowledge, few have clarified the variation of MOMP during metabolism inhibition and the results in cell fate. Herein, we evaluated the mechanism underlying radioresistance that emerged during fractionated radiotherapy to guide the development of MOMP‐modulating liposomes for enhancing CRC radiotherapy. To evaluate the occurrence of radio‐resistance during fractionated radiotherapy of CRC, the downstream proteins of ISR in tumor biopsies from 40 patients were analyzed by immunohistochemistry (IHC). The radioresistance was then simulated in CRC cells to preliminarily confirm the relationship between iMOMP and radioresistance, as well as to screen metabolism‐tailoring drugs for MOMP modulation during radiotherapy. The drug‐loaded liposomes were then developed to circumvent radioresistance and enhance apoptosis of cancer cells under X‐ray irradiations. After confirming the biocompatibility and tumoral accumulation, the liposomes were systematically injected into CRC‐bearing mice to evaluate the enhancement in fractionated radiotherapy and consequential anti‐tumor immune response.

## 2 Results and Discussion

### 2.1 p‐eIF2α and ATF4 Expression Varied in CRC Patients

**Figure 1:**
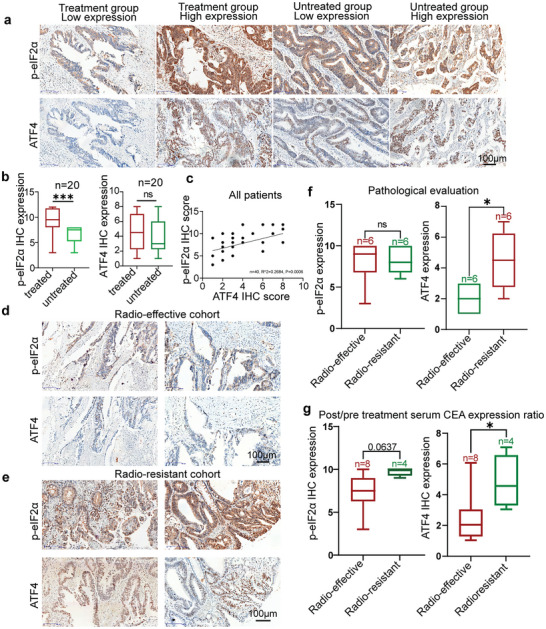
p‐eIF2α and ATF4 expression were significantly upregulated in CRC radio‐resistance patients. a) IHC staining using an antibody against p‐eIF2α and ATF4 and representative photographs of p‐eIF2α and ATF4 in treated and untreated groups of CRC tissues. Scale bar: 100 µm. b) IHC staining score of p‐eIF2α and ATF4 in the 20 pairs of CRC patients. Data were presented as the mean ± SD; statistical significance was assessed by *t*‐test. (^***^ *p* < 0.001, ^**^ *p* < 0.01, ^*^ *p* < 0.05, *n* = 40.) c) Correlation analysis between immunohistochemical staining scores of p‐eIF2α and ATF4 between treated and untreated patients. d) Immunohistochemical staining using antibodies against p‐eIF2α and ATF4, and representative photographs of p‐eIF2α and ATF4 in radiotherapy effective cohort CRC tissues (Scale: 100 µm). **e)** Immunohistochemical staining using antibodies against p‐eIF2α and ATF4, and representative photographs of p‐eIF2α and ATF4 in radio‐resistance cohort CRC tissues. Scale:100 µm. **f)** Statistical analysis p‐eIF2α and ATF4 IHC expression in the resistance cohort and effective cohort by cut‐off of 50% pathological regression or not. Data were presented as the mean ± SD; statistical significance was assessed by t‐test. (^***^ *p* < 0.001, ^**^ *p* < 0.01, ^*^ *p* < 0.05, *n* = 12). **g)** Statistical analysis p‐eIF2α and ATF4 IHC expression in the resistance cohort and effective cohort by post/pre‐treatment serum CEA expression ratio up to 50% or not. Data were presented as the mean ± SD; statistical significance was assessed by *t*‐test. (^***^ *p* < 0.001, ^**^ *p* < 0.01, ^*^ *p* < 0.05, *n* = 12).

It has been reported that p‐eIF2α and ATF4 are the downstream protons of ISR, an intracellular signaling network activated by internal and external stresses, which contribute to cellular escape from apoptosis, therapy tolerance, and tumor invasion and metastasis.^[^ ^27^, ^28^ ^]^ To evaluate the relationship of radiotherapy response with the ISR pathway, we first tested the expression of p‐eIF2α and ATF4 in the biopsies of 40 CRC patients by IHC, which encompasses 20 patients treated by fractionated radiotherapy and 20 patients without radiotherapy (**Figure** **1**a). The IHC analysis demonstrated statistically significant higher scores of p‐eIF2α and generally higher expression of ATF4 in the treated group of CRC tumor slices than the untreated group (Figure 1b). Additionally, the positive correlation between the expression score of p‐eIF2α and ATF4 suggested the activation of the ISR pathway in CRC (Figure 1c).

Moreover, depending on the cut‐off of 50% pathological regression in IHC images, the gold standard for therapeutic efficacy, the treated patients were further classified into radio‐effective and radio‐resistant cohorts. Both p‐eIF2α and ATF4 in radio‐resistant cohorts demonstrated higher expression than those in radio‐effective cohorts (Figure 1d,e), among which ATF4 expression showed statistical difference (Figure 1f). Additionally, the expression ratio of serum carcinoembryonic antigen (CEA) post and preradiotherapy was employed as another standard for the classification of treated patients into radio‐effective and radio‐resistant cohorts, with a cut‐off ratio of 50%. Similarly, the radio‐resistant cohorts demonstrated higher scores of p‐eIF2α and ATF4 than the radio‐sensitive cohorts (Figure 1g). These data collectively confirmed the participation of p‐eIF2α and ATF4 in inducing radioresistance, indicating that the ISR pathway could be targeted to reverse radio resistance in CRC.

### 2.2 Cellular Radio‐Resistance was Mediated by ISR with Upregulated p‐eIF2α and ATF4

**Figure 2:**
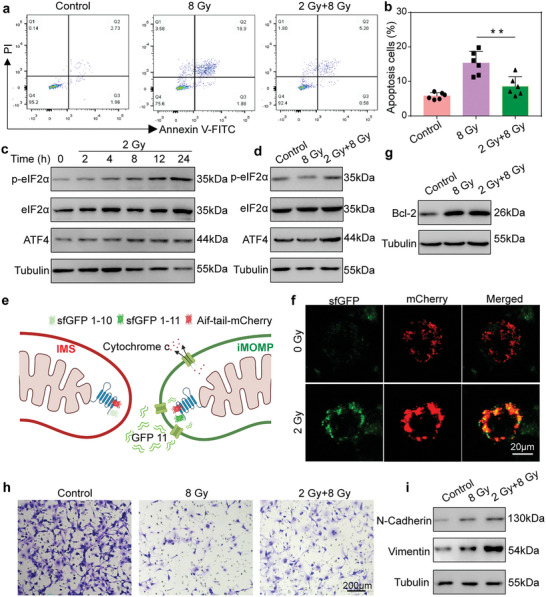
Radio‐resistance induced by X‐ray irradiation. a,b) Flow cytometric analysis of apoptosis in CT26 cells following either 8 Gy or 2+8 Gy irradiations a) and the corresponding statistical apoptosis percentage. Error bars represent mean ± s.d. (*n* = 6). *P* values were calculated by Student's *t*‐tests (^**^ *p* < 0.01). b). c,d) Western blot analysis of p‐eIF2α, eIF2α, and ATF4 expression in CT26 cells after different treatments (Control, 2–24 h after exposure to 2 Gy irradiation) c) and exposed to 8 Gy or 2 Gy +8 Gy irradiations at equivalent time points d). e) Schematic and functional mechanisms of MOMP sensors in mitochondrial and cytoplasmic expression systems. f) Confocal microscopy imaging of CT26 cells overexpressing the MOMP sensor expression system in 0 Gy control group and 2 Gy irradiation group (scale = 20 µm). g) Western blot characterized Bcl‐2 expression in CT26 cells after different treatments (Control, 8 Gy, 2 Gy +8 Gy). h) Transwell Migration Assay experiment to verify the invasiveness of the cells after different treatments (Control, 8 Gy, 2 Gy +8 Gy). i) Western blot analysis of N‐cadherin and Vimentin expression in CT26 cells after different treatments (Control, 2 Gy, 2 Gy+M/D‐Lipo, M/D‐Lipo).

The radioresistance that emerged during fractionated radiotherapy of clinical CRC was then preliminarily investigated by evaluating how the initial X‐ray irradiation influenced the efficacy of radiotherapy in the laboratory. To this end, a simplified simulation of fractionated radiotherapy in vitro was established by treating CT26 cells with consecutive X‐ray irradiations. The flow cytometry results demonstrated less apoptosis ratio in cells exposed to 2 Gy + 8 Gy irradiations than those to 8 Gy (**Figure** **2**a,b), despite the higher total dosage of ionizing radiation. The underlying mechanism contributing to the decrease in apoptosis following repeated X‐ray irradiations was then analyzed. Western blot (WB) experiments were conducted to test the expression of the downstream proteins of ISR in cells after radiotherapy. After a single X‐ray irradiation at 2 Gy, the expression of p‐eIF2α and ATF4 was progressively elevated in CT26 cells until 24 h (Figure 2c). Moreover, the pretreatment of cells by X‐ray irradiation at 2 Gy aggravated the expression of p‐eIF2α and ATF4 (Figure 2d), suggesting the activation of ISR pathway by initial X‐ray exposure in CT26 cancer cells. These results indicated that the initial X‐ray irradiation would activate ISR pathway to upregulate p‐eIF2α and ATF4, preventing cancer cells from apoptosis during the subsequent radiotherapy.

Next, we researched how ISR was activated by X‐ray irradiation. Reports have shown that the extent of MOMP is crucial to cellular fate under mitochondria‐damaging stimuli. MOMP beyond the definitive threshold commits cells to irreversible apoptosis, while iMOMP activates ISR pathway to bypass cell death and even potentiate invasiveness.^[^ ^23^ ^]^ In order to test whether MOMP would be triggered by radiotherapy, a split‐superfold GFP (sfGFP) system was developed to visualize mitochondrial permeability (Figure 2e). In this system, GFP1‐10 was fused to mCherry and targeted to the tail sequence where the fusion intermembrane space (IMS) was located, while the double GFP11β strand was directed to the cytoplasm. Only when MOMP occurs, GFP11 would translocate into the IMS through passive diffusion and subsequently bind to GFP1‐10, resulting in the emission of green fluorescence. CT26 cells with sfGFP system were then monitored by confocal laser scanning microscopy (CLSM). The confocal images of untreated CT26 cells demonstrated the fluorescence of mCherry and no sfGFP (Figure 2f), confirming the success in the transduction of the sfGFP system. In contrast, after the treatment with X‐ray irradiation at 2 Gy, a few CT26 cells demonstrated obvious fluorescence of sfGFP co‐localized with mCherry in mitochondria (Figure 2f), suggesting the induction MOMP of these special cells by a sublethal X‐ray exposure. On the other hand, WB results illustrated that the expression of Bcl‐2 proteins of the cells that survive radiation, which inhibit apoptosis by hampering the mitochondrial membrane permeability to Cyt c,^[^ ^23^ ^]^ was elevated in irradiated cancer cells as compared to the untreated ones (Figure 2g). Especially, CT26 treated with 2 Gy + 8 Gy irradiations demonstrated higher expression of Bcl‐2 proteins than those with 8 Gy (Figure 2g). Therefore, it could be inferred that multiple X‐ray irradiations would upregulate Bcl‐2 proteins in cells, leading to mitochondrial iMOMP and insufficient cytoplasmic Cyt c, resulting in ISR‐mediated radio‐resistance and thus lower apoptosis ratio.

In addition to this, the cells that survive by resisting apoptosis may also possess other malignant characteristics. It has been reported that cancer cells surviving from treatment would not only develop therapy resistance but also raise the tendency toward proliferation and metastasis.^[^ ^10^ ^]^ The clonogenic assay demonstrated that although radiotherapy significantly decreased the number of cell clones, more cells retained the proliferating capability after 2 Gy + 8 Gy irradiations than a single 8 Gy irradiation (FigureS1, Supporting Information). To evaluate the impact of radiotherapy on cellular invasiveness, a transwell migration assay was conducted in CT26 cells after X‐ray irradiations. As compared to cells treated with a single 8 Gy irradiation, 2 Gy + 8 Gy irradiations resulted in a larger amount of CT26 migration (Figure 2h). The number of invasive cells was counted. Compared to cells treated with a single 8 Gy irradiation, 2 Gy + 8 Gy irradiations resulted in larger amount of CT26 migration (Figure S5a, Supporting Information). Moreover, WB results illustrated that the cellular expression of invasiveness‐related proteins including N‐cadherin and vimentin was significantly elevated after 2 Gy + 8 Gy irradiations (Figure 2i). These in vitro results altogether suggested that the mitochondrial iMOMP and ISR‐mediated radio‐resistance should be targeted for addressing the insufficient therapeutic effect and metastasis risk after fractionated radiotherapy.

### 2.3 Drug‐Loaded Liposomes Reversed Radio‐Resistance by Downregulating p‐eIF2α and ATF4

**Figure 3:**
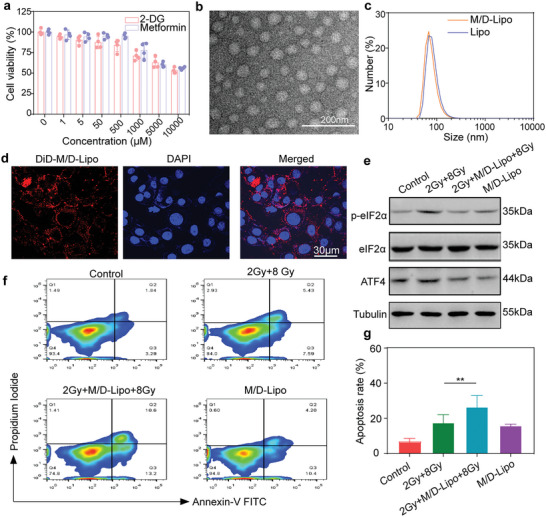
Synthesis and characterization of dual‐drug loaded liposomes. a) The relative cell viability of CT26 cells incubated with metformin or 2‐DG at different concentrations for 24 h. Error bars represent mean ± s.d. (*n* = 6). b) Characterization of M/D‐Lipo by transmission electron microscopy (TEM). c) Hydrodynamic diameters of Lipo and M/D‐Lipo measured by dynamic light scattering (DLS). d) Confocal images of CT26 cells showing the cellular uptake of DIR‐labeled liposomes at 24 h. Red color represents DID signals in liposomes and blue color indicates 4,6‐diamidino‐2‐phenylindole (DAPI) stained cellular nuclei, respectively (scale bar:30 µm). e) Western blot measured p‐eIF2α, eIF2α, ATF4 protein expression in CT26 cells after treated with PBS, 2 Gy +8 Gy, 2 Gy+M/D‐Lipo+8 Gy, M/D‐Lipo. f,g) Flow cytometric analysis of apoptosis f) and statistics g) in CT26 cells after different treatments (*n* = 6). *P* values in g were calculated by Student's *t*‐tests (^*^ *p* < 0.01).

Considering that p‐eIF2α and ATF4 play vital roles in ISR‐mediated radio‐resistance, we then screened drugs that could inhibit the expression of these proteins to address radio‐resistance. Among commercial drugs that are related to mitochondrial functions, Western blots demonstrated that the upregulated p‐eIF2α expression in CT26 cells after 2 Gy irradiation could be partially reversed by metformin, while ATF4 by 2‐DG (Figure S2a, Supporting Information). Interestingly, metformin has been reported to disrupt the function of mitochondria by inhibiting their oxidative respiratory chain.^[^ ^29^ ^]^ Meanwhile, 2‐DG has been demonstrated to inhibit hexokinase 2 (HK‐2), which is a key enzyme in aerobic glycolysis and protects mitochondrial membranes.^[^ ^30^, ^31^ ^]^ Afterward, the toxicity of metformin and 2‐DG to CT26 cells at different concentrations was evaluated by Cell Counting Kit‐8 (CCK8), which demonstrated that 500 µm of each drug is safe in vitro (**Figure** **3**a). Our experimental results, together with earlier reports, indicated that the combination of metformin and 2‐DG may address radio‐resistance in a synergistic manner.

To achieve the simultaneous function, liposomes composed of dipalmitoylphosphatidylcholine (DPPC), cholesterol, and 1, 2‐distearyl phosphatidyleethanolamine‐polyethylene glycol 5000 (DSPE‐PEG 5000) were prepared as a delivery system to load both of metformin and 2‐DG (M/D‐Lipo) (Figure S2b, Supporting Information). It was found that the cell inhibition rate of M/D‐Lipo was equal to the free 2‐DG and metformin (Figure S3a,b, Supporting Information). High‐performance liquid chromatography (HPLC) characterization confirmed the successful loading of the drugs, with a final concentration of 35 mg mL^−1^ metformin and the same concentration of 2‐DG in M/D‐Lipo. Transmission electron microscopy (TEM) imaging showed the spherical morphology of M/D‐Lipo with an average size of 80 nm (Figure 3b). Dynamic light scattering (DLS) demonstrated that the loading of drugs induced negligible variation in the average hydrodynamic diameters (100 nm) and negative potentials (−18.7 mV) (Figure 3c; Figure S4a, Supporting Information). For the convenience of monitoring the cellular uptake of M/D‐Lipo by confocal laser scanning microscopy (CLSM), DiD was then labeled onto the liposomes with stable fluorescence (Figure S4b, Supporting Information). After the incubation with CT26 cells for 24 h, confocal imaging demonstrated that the DiD‐Lipo was endocytosed into the cytoplasm of the tumor cells (Figure 3d), which guaranteed the drug delivery capacity.

The mechanism was further analyzed by WB detection of p‐eIF2α and ATF4 expression. The WB assays revealed that while 2 Gy + 8 Gy irradiations induced the upregulation of p‐eIF2α and ATF4 in CT26 cells, M/D‐Lipo reversed this progress and inhibited the expression of proteins concerning radio‐resistance (Figure 3e). The influence of M/D‐Lipo on radiotherapy was then evaluated by exposing CT26 cells to X‐ray irradiation with or without M/D‐Lipo. Flow cytometry analysis of cells after different treatments showed that the addition of M/D‐Lipo during multiple irradiations could elevate the apoptosis ratio of cells (Figure 3f,g). Altogether, M/D‐Lipo reversed radio‐resistance and promoted cellular apoptosis by modulating the downstream and upstream proteins in the resistant pathway initiated by nonlethal mitochondria damage. These results inspired us to further evaluate the morphology and functions of mitochondria in cells treated with X‐ray irradiations and M/D‐Lipo.

### 2.4 M/D‐Lipo Decreased MMP, Reprogram Metabolism, and Inhibit Invasiveness of Cancer Cells

**Figure 4:**
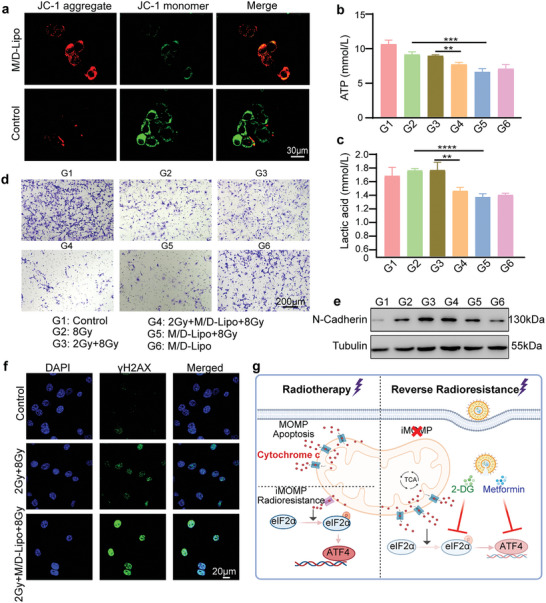
M/D‐Lipo reversal of radio‐resistance. a) Confocal laser scanning microscope (CLSM) images of CT26 cells treated with PBS and M/D‐Lipo for 24 h after staining with JC‐1 probe for 30 min. (scale bar: 30 µm) b) ATP production experiment showed the production of ATP in the cytoplasm of CT26 cells after different treatments. Error bars represent mean ± s.d. (*n* = 6). c) Lactate production experiments showed that CT26 cells produced lactate after different treatments. Error bars represent mean ± s.d. (*n* = 6). d) Transwell migration assay demonstrated the invasiveness of CT26 cells after different treatments. (scale bar: 200 µm.) e) The invasion‐associated protein N‐Cadherin detected by Western blot was consistent with the results verified by the transwell migration assay. f) Immunofluorescent images showing the expression of γH2AX protein in CT26 cells with different treatments. (scale bar:20 µm). g) Schematic representation of the mechanisms by which M/D‐Lipo altered tumor radio‐sensitivity, radio‐resistance, and metabolic reprogramming. Treatments: PBS (G1), 8 Gy (G2), 2 Gy+8 Gy (G3), 2 Gy+M/D‐Lipo+8 Gy (G4), M/D‐Lipo+8 Gy (G5), M/D‐Lipo (G6). *P* values in b and c were calculated by Student's *t*‐tests (^****^ *p* < 0.0001,^***^ *p* < 0.001, ^**^ *p* < 0.01).

Normal mitochondrial membrane potential (MMP) is a prerequisite for maintaining mitochondrial functions, while the decline of MMP is an early event of apoptosis.^[^ ^32^ ^]^ JC‐1 probe was then applied to monitor the influence of M/D‐Lipo on MMP in CT26 cells. The confocal images demonstrated that, as compared to the control group, the cells treated with M/D‐Lipo showed weaker red fluorescence for JC‐1 aggregates and stronger green fluorescence for JC‐1 monomer (**Figure** **4**a), indicating the decline of MMP by M/D‐Lipo. Next, the metabolism status of cells after different treatments was evaluated by testing the levels of adenosine triphosphate (ATP) and lactic acid. The data demonstrated that either single or double X‐ray irradiations slightly decreased the production of ATP in cells (Figure 4b), while the inhibition of ATP production was further enhanced by M/D‐Lipo which contained metformin suppressing oxidative phosphorylation.^[^ ^33^ ^]^ On the other hand, double X‐ray irradiations induced more lactic acid in cells than single exposure (Figure 4c), indicating the more severe hypoxia by multiple radiotherapy treatments. Meanwhile, the treatment of cells with M/D‐Lipo in all circumstances induced a substantial decrease in the lactic acid level (Figure 4c), which could be ascribed to 2‐DG inhibiting the pivotal enzyme (HK2) in the glycolysis pathway producing lactic acid.^[^ ^34^ ^]^ These results confirmed the function of M/D‐Lipo on the mitochondria of cancer cells, resulting in MMP decline and metabolic reprogramming with decreased ATP and lactic acid, which would facilitate cellular apoptosis by radiotherapy.

Moreover, the invasiveness of CT26 cells treated with M/D‐Lipo and X‐ray irradiations was evaluated by a transwell migration assay and WB analysis. In accordance with previous results, double irradiations by X‐ray increased the invasiveness of CT26 cells (Figure 4d). Compared to 8 Gy or 2 Gy + 8 Gy irradiations, the 2 Gy+M/D‐Lipo+8 Gy and M/D‐Lipo+8 Gy resulted in less migration (Figure 4d; Figure S5b, Supporting Information). The same results were confirmed by the cell scratch assay (Figure S6a–c, Supporting Information). Moreover, WB analysis of N‐cadherin, which is attributed to cell separation and motility, demonstrated that the increase of N‐cadherin by X‐ray irradiation was mitigated by M/D‐Lipo (Figure 4e), which was consistent with the changes in cell invasion ability revealed by the transwell experiments. These results suggested that M/D‐Lipo with the capability of modulating mitochondria could not only reverse radio‐resistance to enhance apoptosis but also hamper the invasion tendency of residual cancer cells. The immunofluorescent imaging also demonstrated the most obvious gH2AX foci in cells treated with the combination of radiotherapy and M/D‐Lipo (Figure 4f). These results confirmed that the nuclear damage was reduced during radio‐resistance. However, when the radio resistance was reversed by M/D‐Lipo, the nuclear damage and apoptosis were increased. Therefore, we illustrated the mechanisms of radiotherapy resistance and drug resistance in cancer cells through schematic diagrams (Figure 4g).

### 2.5 Radiotherapy of CRC in Vivo was Enhanced by M/D‐Lipo

**Figure 5:**
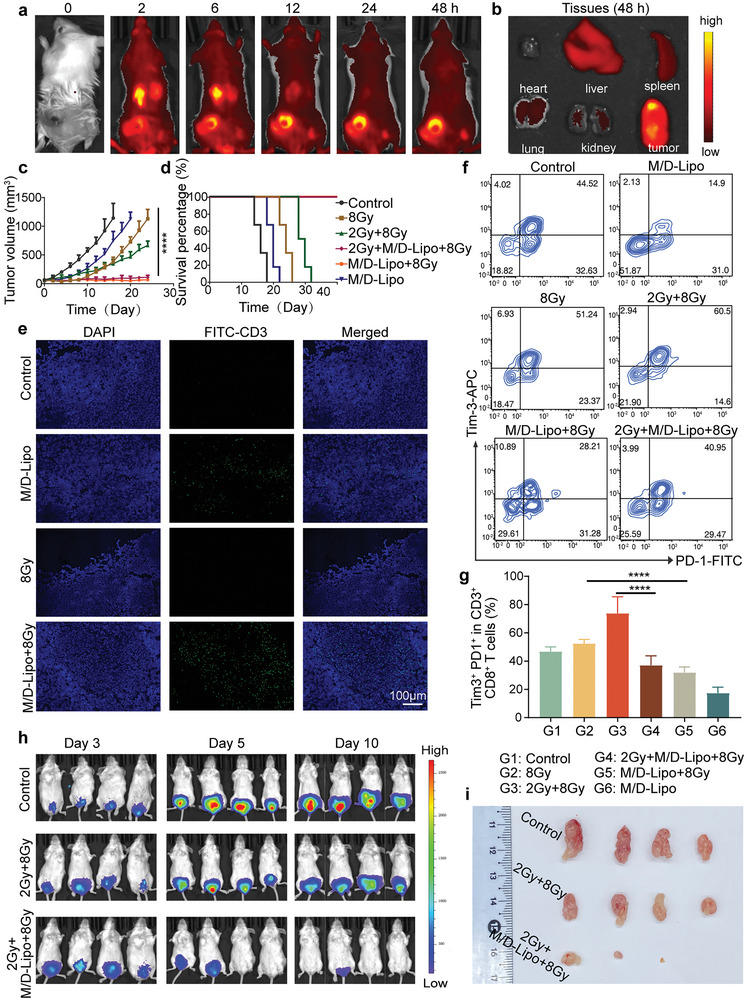
M/D‐Lipo combined with RT could enrich T cells at the tumor site. a) The fluorescence imaging of mice by IVIS at different time points after intravenous injection of DIR‐labelled M/D‐Lipo. b) The ex vivo fluorescence imaging of organs and tumors collected at 48 h post systemic administration. c) The tumor volume curves of mice post different treatments (PBS, 2 Gy+8 Gy, 8 Gy, M/D‐Lipo, 2 Gy+M/D‐Lipo+8 Gy, M/D‐Lipo+8 Gy) (*n* = 6 in each group). d) Survival curves of mice post different treatments. (e) Immunofluorescence staining showed CD3^+^ T lymphocytes (green) in the subcutaneous tumor after 5 days of different treatments (scale bar:100 µm). f,g) Flow cytometry analysis of subcutaneous tumors f) and statistics Error bars represent mean ± s.d. (*n* = 6). g) revealed a reduction in the number of exhausted T cells in the tail vein injection of M/D‐Lipo group, indicating that the combination could reverse the exhaustion of T cells in the immune microenvironment. h) Monitoring CT26 colorectal cancer growth and metastasis after treatment with M/D‐Lipo and/or radiotherapy using in vivo bioluminescence imaging (*n* = 4). i) Photographs of orthotopic tumors from mice in different treatment groups Error bars represent mean ± s.d. (*n* = 4). *p* values in c and g were calculated by Student's *t*‐tests (^****^ *p* < 0.0001).

Before evaluating the effect of M/D‐Lipo on radiotherapy of CT26 tumor‐bearing mice, the biosafety was assessed by hematoxylin and eosin (H&E) staining major organs and tissues collected from mice at 7 days after the intravenous (i.v.) injection of M/D‐Lipo (Figure S7, Supporting Information). The histopathological analysis preliminarily demonstrated that M/D‐Lipo had no observable toxicity to mice. Next, the biodistribution and tumor accumulation behavior of M/D‐Lipo were monitored by an in vivo imaging system (IVIS) at different time points after i.v. injection of DiR‐labeled M/D‐Lipo into the mice. The tumors showed obvious fluorescence at 2 h postinjection, which signals intensity increased along with time until 12 h and remained strong even after 48 h (**Figure** **5**a). Meanwhile, the fluorescence of DiR‐labeled M/D‐Lipo gradually faded in the abdomen (Figure 5a). Afterward, mice were sacrificed to collect the major organs and tumors for ex vivo imaging. Consistent to the IVIS imaging of mice, the fluorescence imaging showed high accumulation of M/D‐Lipo in tumors and less in the liver and spleen (Figure 5b) and fluorescence statistics showed high accumulation of M/D‐Lipo in tumors and less in liver and spleen (Figure S8, Supporting Information). These results confirmed the biosafety and tumor accumulation capability of M/D‐Lipo, suggesting its potential for tumor therapy.

The subcutaneous CRC model was established by injecting CT26 cells in Balb/c mice. After the tumor volume achieved 50 mm^3^, the tumor‐bearing mice were randomly categorized into six groups and monitored every 2 days. The tumor volume curves demonstrated the X‐ray exposure at 8 Gy slowed down the growth of tumors during the 24‐day observation period (Figure 5c). Meanwhile, 2 Gy + 8 Gy X‐ray irradiations inhibited the tumors in the first 8 days while growing rapidly afterward (Figure 5c). Additionally, none of the mice in these two radiotherapy groups survived 24 days after the treatments (Figure 5c). These data confirmed the incapability of radiotherapy alone to eradicate tumors and the risk of fractionated radiotherapy in elevating cancer proliferation. The administration of M/D‐Lipo retarded the tumor growth rate but did not eliminate tumors or prolong survival sufficiently compared to the control group (Figure 5c). Encouragingly, the combination of M/D‐Lipo with radiotherapy either at the dosage of 8 Gy or 2 Gy + 8 Gy could completely eliminate the tumors and prolong the survival time of mice to longer than 40 days (Figure 5c,d). Immunofluorescence staining of p‐eIF2α and ATF4 in tumor tissues of mice after 5 days of treatment showed that the expression of p‐eIF2α and ATF4 was increased after 2 Gy + 8 Gy radiotherapy, while M/D‐Lipo could down‐regulate the expression (Figure S9, Supporting Information). There was no significant difference in body weight between the groups. (Figure S10, Supporting Information).

The satisfactory therapeutic efficacy of the combination of M/D‐Lipo and radiotherapy encouraged us to further research the immune environment of tumors after each treatment. The treatment with M/D‐Lipo has been demonstrated to augment the distribution of CD3^+^ T cells within the tumor site (Figure 5e), aligning with reported findings on the potential of metformin to enhance anti‐tumor immune effects.^[^ ^31^ ^]^ Additionally, flow cytometric analysis of Tim‐3^+^ PD‐1^+^ T cells in tumors 1‐week post‐treatment revealed proportions in the control group were 46.85 ± 3.25%, 8 Gy group was 52.52 ± 2.87%, 2 Gy+8 Gy group was 73.80 ± 11.71%, 2 Gy+M/D‐Lipo+8 Gy group was 37.02 ± 6.75%, 8 Gy+M/D‐Lipo group was 31.95 ± 3.97%, and M/D‐Lipo group was 17.44 ± 4.17% (Figure 5f,g). Importantly, M/D‐Lipo demonstrated the ability to decrease the proportion of Tim‐3^+^ PD‐1^+^ T cells, thereby further reducing T cell exhaustion and depletion within the tumor tissue following radiation. This suggested that metformin can activate endogenous immunity by suppressing T‐cell exhaustion.

Moreover, the tumor in situ model has a greater resemblance to the actual in vivo scenario, thus rendering it a more realistic tool for assessing the efficacy of anti‐tumor medications. In order to better illustrate the problem, we next constructed a low rectal cancer model in BALB/C mice using CT26 cell line (Figure S11, Supporting Information). Tumor growth and treatment could be observed on the 7th day after tumor implantation, and the therapeutic effect was observed by IVIS on the 3rd, 5th, and 10th day after treatment. The results showed that the combination therapy had a better therapeutic effect than radiotherapy alone (Figure 5h). At the end of treatment, the mice were dissected, and the colorectal tumors were taken out for photographs (Figure 5i) and weighed (Figure S12a, Supporting Information). In 2 Gy+M/D‐Lipo+8 Gy group (G3), the tumor was basically reduced or cured. In addition, H&E sections from the small intestine to the anus showed significant shrinkage of tumor tissue after combined treatment with 2 Gy+M/D‐Lipo+8 Gy (G3), while in the control group, the tumors continued to grow with necrosis in the center (Figure S12b, Supporting Information). Staining for PD‐L1 in tissue‐frozen sections on day 3 after radiotherapy showed that radiotherapy significantly increased PD‐L1 expression, which was effectively inhibited by M/D‐Lipo treatment, reducing PD‐L1 levels in tumor tissue (Figure S13, Supporting Information).

### 2.6 M/D‐Lipo Increased Immune Effect of Radiotherapy by Reducing T Cell Depletion

**Figure 6:**
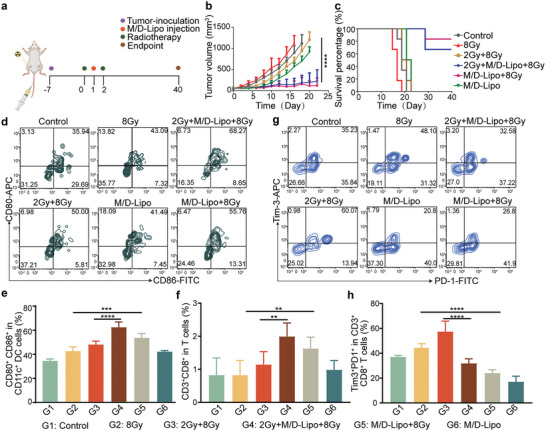
M/D‐lipo enhanced the abscopal effect of radiotherapy. a) Schematic diagram of the local tumor treatment protocol in mice. b) Tumor volume curves in mice after different treatments (PBS, 2 Gy+8 Gy, 8 Gy, M/D‐Lipo, 2 Gy+M/D‐Lipo+8 Gy, M/D‐Lipo+8 Gy) (*n* = 6 in each group). c) Survival curves of mice after different treatments. d,e) Representative flow cytometry d) and the quantified plots e) showing the amount of matured DCs in lymph nodes (LNs) from mice 5 days post each treatment (*n* = 6). (f) The accumulation of T cells in the distal metastases of mice induced by the above treatments was measured by flow cytometry. g,h) FACS plots g) and Statistic data h) of Tim‐3^+^ T cell by above‐mentioned treatments on mice. Error bars represent mean ± s.d. (*n* = 6). *p* values in b,e, f, and h were calculated by Student's *t*‐tests (^****^ *p* < 0.0001,^***^ *p* < 0.001, ^**^ *p* < 0.01).

Subsequently, we established a bilateral tumor model and administered local radiotherapy 1 day before (2 Gy) and after (8 Gy) tail intravenous injection of M/D‐Lipo (**Figure** **6**a). Monitoring the tumor growth curve showed partial suppression of tumors in the 2 Gy+M/D‐Lipo+8 Gy group (survival rate at endpoint, 66.7%) and entire suppression of tumors in the M/D‐Lipo+8 Gy (survival rate at endpoint, 83.3%) groups (Figure 6b). In contrast to the control group (median survival, 19 days), simple 8 Gy treatment exhibited a little increase in tumor growth rate and a decrease in overall survival (median survival, 20 days). In the 2 Gy + 8 Gy group, the inhibitory effect on distant tumors was not obvious, with a median survival time of 17 days, while in the M/D‐Lipo group, the inhibitory effect on distant tumors was obvious, with a median survival time of 22 days (Figure 6c). Besides, there was no significant difference in body weight between the groups (Figure S14, Supporting Information).

In order to ascertain the underlying mechanism of this phenomenon, we focused on the infiltration of immune cells originating from the distant tumor site. In the mice, distal tumor model, comparison to PBS, immune cell analysis conducted after 5 days of different treatments revealed that radiotherapy promoted the maturation of DC cells in tumor‐associated lymph nodes, while M/D‐Lipo further increased the effect of this enhancement (Figure 6d,e). This might be the key point to increase the distant effects of radiotherapy. The combination of M/D‐Lipo and radiation resulted in an increase in the overall fraction of CD3^+^ CD8^+^ T cells within the distant tumor (Figure 6f). What's more, the introduction of M/D‐Lipo led to a notable reduction in the percentage of exhausted T cells. Specifically, the percentage of exhausted T cells in the control group was 37.11% ± 1.52%, whereas the percentage of exhausted T cells in the M/D‐Lipo group was 17.08% ± 4.76%. Similarly, the percentage in the 8 Gy group was 45.00% ± 3.42% compared to 24.12% ± 2.74% in the M/D‐Lipo+8 Gy group. In the case of the 2 Gy + 8 Gy group, a proportion of 57.51% ± 8.56% was observed, and the corresponding 2 Gy+M/D‐Lipo+8 Gy group displayed a statistically significant decrease 31.97% ± 3.73% (Figure 6g,h). This may be due to the ability of metformin to protect CD8‐positive T cells from hypoxia‐induced apoptosis and immunosuppression.^[^ ^35^ ^]^ Notably, even in the absence of direct irradiation, there was a significant up‐regulation of exhausted T cells in the 2 Gy +8 Gy group compared to the control group, which highlights an intriguing phenomenon whose underlying mechanism is unclear and warrants further investigation.

## 3 Conclusion

In summary, we obtained a total of 40 samples of colorectal cancer, consisting of 20 samples from patients who received fractionated radiotherapy and 20 samples from patients who did not receive radiotherapy. Our observation revealed that the expression levels of p‐eIF2α and ATF4 were consistently elevated and positively correlated in colorectal cancer samples after radiotherapy. Cellular investigations confirmed that a single dose of radiation‐induced an elevation in iMOMP in cancer cells, resulting in a significant upregulation of p‐eIF2α and ATF4. This process facilitated the development of resistance to later radiation, hence diminishing its efficacy and potentially augmenting the invasive properties of certain tumor cells. To address this issue, we developed a delivery method utilizing the FDA‐approved drugs metformin and 2‐DG encapsulated in PEGylated liposomes. This system efficiently delivered sufficient concentrations of drugs to the tumor. Animal studies exhibited that these drug‐containing liposomes not only reversed the resistance generated by a single dose of radiotherapy but also altered the state of T‐cell exhaustion in the tumor microenvironment. This modification improved the radiotherapy‐induced immune response, resulting in a long‐term anti‐tumor effect. In summary, we proposed a hypothetical mechanism for the development of radiotherapy resistance in specific tumor cells during fractionated radiotherapy. This resistance was believed to be caused by the activation of intrinsic mitochondrial outer membrane permeabilization (iMOMP) after a single radiotherapy session. Therefore, we have designed and validated the efficacy of liposomal drug formulations targeting this mechanism, providing a viable strategy to address the poor efficacy of fractionated radiotherapy in the clinic.

## Conflict of Interest

The authors declare no conflict of interest.

## Supporting information

Supporting Information

## Data Availability

The data that support the findings of this study are available on request from the corresponding author. The data are not publicly available due to privacy or ethical restrictions.

## References

[1] F. Roeder , E. Meldolesi , S. Gerum , V. Valentini , C. Rodel , Radiat. Oncol. 2020, 15, 262. 33172475 10.1186/s13014-020-01695-0 PMC7656724

[2] M. M. Center , A. Jemal , R. A. Smith , E. Ward , CA Cancer J. Clin. 2009, 59, 366. 19897840 10.3322/caac.20038

[3] A. Shaukat , T. Kaltenbach , J. A. Dominitz , D. J. Robertson , J. C. Anderson , M. Cruise , C. A. Burke , S. Gupta , D. Lieberman , S. Syngal , D. K. Rex , Gastroenterology 2020, 159, 1916. 33159840 10.1053/j.gastro.2020.08.050

[4] N. Gupta , S. S. Kupfer , A. M. Davis , JAMA, J. Am. Med. Assoc. 2019, 321, 2022. 10.1001/jama.2019.4842 PMC7285652 31021387

[5] J. P. Ryuk , G. S. Choi , J. S. Park , H. J. Kim , S. Y. Park , G. S. Yoon , S. H. Jun , Y. C. Kwon , Ann. Surg. Treat. Res. 2014, 86, 143. 24761423 10.4174/astr.2014.86.3.143 PMC3994626

[6] A. M. Abulafi , N. S. Williams , Brit. J. Surg. 1994, 81, 7.8313126

[7] S. Sheikh , H. Chen , A. Sahgal , I. Poon , D. Erler , S. Badellino , R. Dagan , M. C. Foote , A. V. Louie , K. J. Redmond , U. Ricardi , T. Biswas , Radiother. Oncol. 2022, 167, 187. 34952002 10.1016/j.radonc.2021.12.018

[8] G. Feeney , R. Sehgal , M. Sheehan , A. Hogan , M. Regan , M. Joyce , M. Kerin , World J. Gastroenterol. 2019, 25, 4850. 31543678 10.3748/wjg.v25.i33.4850 PMC6737323

[9] R. Zheng , S. Zhang , H. Zeng , S. Wang , K. Sun , R. Chen , L. Li , W. Wei , J. He , J. Nat. Cancer Center 2022, 2, 1. 10.1016/j.jncc.2022.02.002 PMC11256658 39035212

[10] Y. Pu , L. Li , H. Peng , L. Liu , D. Heymann , C. Robert , F. Vallette , S. Shen , Nat. Rev. Clin. Oncol. 2023, 20, 799. 37749382 10.1038/s41571-023-00815-5

[11] C. Ye , X. Zhang , J. Wan , L. Chang , W. Hu , Z. Bing , S. Zhang , J. Li , J. He , J. Wang , G. Zhou , Cell Cycle 2013, 12, 1424. 23574719 10.4161/cc.24528 PMC3674070

[12] V. C. Banwell , H. A. Phillips , M. J. Duff , D. Speake , C. McLean , L. J. Williams , Y. He , H. M. Paterson , Acta Oncol. 2019, 58, 1267. 31237192 10.1080/0284186X.2019.1631473

[13] J. F. Bosset , L. Collette , G. Calais , L. Mineur , P. Maingon , L. Radosevic‐Jelic , A. Daban , E. Bardet , A. Beny , J. C. Ollier , E. R. G. Trial , N. Engl. J. Med. 2006, 355, 1114. 16971718 10.1056/NEJMoa060829

[14] N. N. Rahbari , H. Elbers , V. Askoxylakis , E. Motschall , U. Bork , M. W. Buchler , J. Weitz , M. Koch , Ann. Surg. Oncol. 2013, 20, 4169. 24002536 10.1245/s10434-013-3198-9

[15] G. A. Higgins, Jr. , J. H. Conn , P. H. Jordan, Jr. , E. W. Humphrey , B. Roswit , R. J. Keehn , Ann. Surg. 1975, 181, 624. 805571 10.1097/00000658-197505000-00017 PMC1345553

[16] M. Han , E. A. Bushong , M. Segawa , A. Tiard , A. Wong , M. R. Brady , M. Momcilovic , D. M. Wolf , R. Zhang , A. Petcherski , M. Madany , S. Xu , J. T. Lee , M. V. Poyurovsky , K. Olszewski , T. Holloway , A. Gomez , M. S. John , S. M. Dubinett , C. M. Koehler , O. S. Shirihai , L. Stiles , A. Lisberg , S. Soatto , S. Sadeghi , M. H. Ellisman , D. B. Shackelford , Nature 2023, 615, 712. 36922590 10.1038/s41586-023-05793-3 PMC10033418

[17] A. S. Monzel , J. A. Enriquez , M. Picard , Nat. Metab. 2023, 5, 546. 37100996 10.1038/s42255-023-00783-1 PMC10427836

[18] J. E. Chipuk , G. P. McStay , A. Bharti , T. Kuwana , C. J. Clarke , L. J. Siskind , L. M. Obeid , D. R. Green , Cell 2012, 148, 988. 22385963 10.1016/j.cell.2012.01.038 PMC3506012

[19] X. Liu , R. Fu , Y. Pan , K. F. Meza‐Sosa , Z. Zhang , J. Lieberman , Cell 2018, 174, 187. 29779946 10.1016/j.cell.2018.04.017

[20] P. Zheng , B. Ding , R. Shi , Z. Jiang , W. Xu , G. Li , J. Ding , X. Chen , Adv. Mater. 2021, 33, 2007426. 10.1002/adma.202007426 33675268

[21] P. Zheng , J. Ding , Asian J Pharm. Sci. 2022, 17, 1. 35261641 10.1016/j.ajps.2021.10.004 PMC8888138

[22] P. Zheng , B. Ding , Z. Jiang , W. Xu , G. Li , J. Ding , X. Chen , Nano Lett. 2021, 21, 2088. 33596078 10.1021/acs.nanolett.0c04778

[23] H. Kalkavan , M. J. Chen , J. C. Crawford , G. Quarato , P. Fitzgerald , S. W. G. Tait , C. R. Goding , D. R. Green , Cell 2022, 185, 3356. 36055199 10.1016/j.cell.2022.07.025 PMC9450215

[24] C. R. Bartman , B. Faubert , J. D. Rabinowitz , R. J. DeBerardinis , Nat. Rev. Cancer 2023, 23, 863. 37907620 10.1038/s41568-023-00632-z PMC11161207

[25] W. Shen , T. Liu , P. Pei , J. Li , S. Yang , Y. Zhang , H. Zhou , L. Hu , K. Yang , Adv. Mater. 2022, 34, 2207343. 10.1002/adma.202207343 36222379

[26] G. J. Yoshida , J. Exp. Clin. Cancer Res. 2015, 34, 111. 26445347 10.1186/s13046-015-0221-y PMC4595070

[27] J. M. Winter , T. Yadav , J. Rutter , Mol. Cell 2022, 82, 3321. 35961309 10.1016/j.molcel.2022.07.012 PMC9481690

[28] T. Verfaillie , A. D. Garg , P. Agostinis , Cancer Lett. 2013, 332, 249. 20732741 10.1016/j.canlet.2010.07.016

[29] S. Soberanes , A. V. Misharin , A. Jairaman , L. Morales‐Nebreda , A. C. McQuattie‐Pimentel , T. Cho , R. B. Hamanaka , A. Y. Meliton , P. A. Reyfman , J. M. Walter , C. I. Chen , M. Chi , S. Chiu , F. J. Gonzalez‐Gonzalez , M. Antalek , H. Abdala‐Valencia , S. E. Chiarella , K. A. Sun , P. S. Woods , A. J. Ghio , M. Jain , H. Perlman , K. M. Ridge , R. I. Morimoto , J. I. Sznajder , W. E. Balch , S. M. Bhorade , A. Bharat , M. Prakriya , N. S. Chandel , et al., Cell Metab. 2019, 29, 335. 30318339 10.1016/j.cmet.2018.09.019 PMC6365216

[30] B. Pajak , E. Siwiak , M. Soltyka , A. Priebe , R. Zielinski , I. Fokt , M. Ziemniak , A. Jaskiewicz , R. Borowski , T. Domoradzki , W. Priebe , Int. J. Mol. Sci. 2019, 21, 223. 31905745 10.3390/ijms21010234 PMC6982256

[31] X. Huang , T. Sun , J. Wang , X. Hong , H. Chen , T. Yan , C. Zhou , D. Sun , C. Yang , T. Yu , W. Su , W. Du , H. Xiong , Cancer Res. 2023, 83, 2358. 37195082 10.1158/0008-5472.CAN-22-3042

[32] S. Bonnet , S. L. Archer , J. Allalunis‐Turner , A. Haromy , C. Beaulieu , R. Thompson , C. T. Lee , G. D. Lopaschuk , L. Puttagunta , S. Bonnet , G. Harry , K. Hashimoto , C. J. Porter , M. A. Andrade , B. Thebaud , E. D. Michelakis , Cancer Cell 2007, 11, 37. 17222789 10.1016/j.ccr.2006.10.020

[33] H. Xian , Y. Liu , A. Rundberg Nilsson , R. Gatchalian , T. R. Crother , W. G. Tourtellotte , Y. Zhang , G. R. Aleman‐Muench , G. Lewis , W. Chen , S. Kang , M. Luevanos , D. Trudler , S. A. Lipton , P. Soroosh , J. Teijaro , J. C. de la Torre , M. Arditi , M. Karin , E. Sanchez‐Lopez , Immunity 2021, 54, 1463. 34115964 10.1016/j.immuni.2021.05.004 PMC8189765

[34] Y. S. Wijayasinghe , M. P. Bhansali , M. R. Borkar , G. U. Chaturbhuj , B. S. Muntean , R. E. Viola , P. R. Bhansali , J. Med. Chem. 2022, 65, 3706. 35192360 10.1021/acs.jmedchem.1c01737

[35] V. Finisguerra , T. Dvorakova , M. Formenti , P. Van Meerbeeck , L. Mignion , B. Gallez , B. J. , J. Immunother. Cancer 2023, 11, e005719. 37147018 10.1136/jitc-2022-005719 PMC10163559

