# Supplementary material for: Mitochondria‐Modulating Liposomes Reverse Radio‐Resistance for Colorectal Cancer
Source: Adv Sci (Weinh). 2024 Mar 23;11(18):2400845. doi: 10.1002/advs.202400845 (PMC11095197; doi:10.1002/advs.202400845)
Supplement: Supplementary file 1 — Supporting Information [file ADVS-11-2400845-s001.pdf]

ADVANCED SCIENCE
Open Access

Supporting Information

for *Adv. Sci.*, DOI 10.1002/advs.202400845

Mitochondria-Modulating Liposomes Reverse Radio-Resistance for Colorectal Cancer

Junmei Li, Yuhong Wang, Wenhao Shen, Ziyu Zhang, Zhiyue Su, Xia Guo, Pei Pei, Lin Hu, Teng Liu, Kai Yang* and Lingchuan Guo**

Supplementary Materials

Mitochondria-Modulating Liposomes Reverse Radio-resistance for Colorectal Cancer

Junmei Li[#], Yuhong Wang[#], Wenhao Shen[#], Ziyu Zhang, Zhiyue Su, Xia Guo, Pei Pei, Lin Hu, Teng Liu[*], Kai Yang[*] Lingchuan Guo[*]

Prof. K. Yang, Prof. L.C. Guo, Miss. J.M. Li., Mrs. Y.H. Wang, Dr. Z.Y. Su, Mrs. X. Guo,
Department of Pathology, the First Affiliated Hospital of Soochow University, Soochow University, Suzhou 215123, Jiangsu, China.

Prof. K. Yang, Prof. T. Liu, Miss. Z.Y. Zhang, Dr. P. Pei, Prof. L. Hu,
State Key Laboratory of Radiation Medicine and Protection, School of Radiation Medicine and Protection & School for Radiological and Interdisciplinary Sciences (RAD-X), Collaborative Innovation Center of Radiation Medicine of Jiangsu Higher Education Institutions, Suzhou Medical College, Soochow University, Suzhou 215123, Jiangsu, China.

Dr. W.H. Shen
Department of Oncology, Taizhou People's Hospital Affiliated to Nanjing Medical University, Taizhou 225300, China.

[#]J. L., Y. W. and W. S. contributed equally to this work.
[*]Corresponding Authors: tliu13@suda.edu.cn; kyang@suda.edu.cn; szglc@hotmail.com.

Materials and Methods

Materials

2-Deoxy-D-Glufcose was purchased from Adamas-beta Co., Ltd. Metformin Hydrochloride was purchased from Adamas-beta Co., Ltd. ATP Assay Kit was purchased from Beyotime Co., Ltd. Lactic Acid (LA) Content Assay Kit was purchased from Solarbio Co., Ltd. Mitochondrial Membrane Potential Assay Kit with JC-1 was purchased from Beyotime Co., Ltd. In Vivo MAb anti-mouse PD-L1 was purchased from Bio X Cell (USA). The primary antibodies including p-eIF2α (Zen Bio), eIF2α (Zen Bio), ATF4 (Abcam), Bcl2 (Abcam), N-Cadherin (Abcam), Vimentin (Abcam), Beta tublin (Abcam). Anti-CD86-APC (eBioscience), anti-CD80-PE (eBioscience), anti-CD11c-FITC (eBioscience), anti-CD8-PE (eBioscience), anti-CD3-FITC (eBioscience), anti-PD-1-FITC (eBioscience), anti-Tim3-APC (eBioscience) were purchased from Zvxcbio (Suzhou, China). Cell culture vessels were purchased from NEST (Wuxi, China). DMEM (high glucose, Gibco), penicillin/streptomycin (P/S) (New Cell & Molecular Biotech Co., Ltd. (Suzhou, China)) and fetal bovine serum (FBS) (NCM Biotech (Suzhou, China)) were purchased from Yibang Biotechnology Co., Ltd. (Suzhou, China).

Methods

Generation of stable CT26 cell lines expressing MOMP sensor system

Firstly, all coding sequences of MOMP sensor system were directly synthesized (GENEWIZ). The hAIF-myc-mCherry-GFP1-10 sequence was subcloned into a pCDH-CMV-MCS-EF1-Puro vector using the endonucleases EcoRI/NotI (New England Biolabs, NEB; # R0101S/R0189S) to generate the GFP1-10 expression vector. Similarly, the GFP11 was subcloned into a pCDH-CMV-MCS-EF1-Zeocin vector with the endonucleases EcoRI/NotI to yield the GFP11 expression vector. Then, the lentiviral vectors GFP1-10 and GFP11 were respectively co-transfected with packaging plasmids psPAX2 and pMD2.G (RIBOBIO) into HEK 293T cells using Lipofectamine 3000 (Invitrogen, #L3000015) for 48 h. Packaged lentiviruses were collected and used to infect CT26 cells for 72 h. Finally, The CT26 cells expressing MOMP sensor system were selected with 1 ug/mL puromycin (Solarbio, #P8230) and 1 ug/mL Zeocin™ (Invitrogen) .

Immunohistochemistry (IHC)

The expression levels of p-eIF2α and ATF4 were assessed using IHC on paired paraffin-preserved tissue sections from 20 patients with CRC and 20 patients with CFR. Immunohistochemistry was performed on 2 μm sections using the BenchMark ULTRA automated stainer (Ventana Medical Systems, Inc., Tucson, Arizona, USA) in accordance with the manufacturer’s protocols. Primary p-eIF2α and ATF4 antibody was obtained from zenbio (#381426/#310073, anti-p-eIF2α and ATF4 diluted 1:50). Each specimen was scored according to the proportion of positive cancer cells as follows: 1, 0–25%; 2, 25–50%; 3, 50–75%; and 4, > 75%. Specimens were also scored according to the staining intensity of cancer cells as follows: 0, negative; 1, light yellow; 2, dark yellow; 3, brown. The IHC staining score was calculated by multiplying the proportion of positive cancer cells by the staining intensity of cancer cells. The staining results were evaluated by two independent pathologists who had at least 5 years working experience. All samples were obtained with approval from the Institutional Ethics Committee of the First Affiliated Hospital of Soochow University (authorisation number 2021333).

Preparation of the drug delivery system (DDS)

Dipalmitoylphosphatidylcholine (DPPC) (Ruixibio Co., Ltd. (Xi’an, China)), cholesterol (J&K Scientific (Beijing, China)), and 1,2-distearoylphosphatidylethanolamine-polyethylene glycol 5000 (DSPE-PEG5000) (Ruixibio Co., Ltd. (Xi’an, China)) were dissolved in chloroform at a molar ratio of 6:4:0.5. The solution was dried by rotary evaporator at 40 °C to produce liposomes. Afterwards, M/D-Lipo was prepared by adding metformin hydrochloride (Meron Bio, Dalian, China) and 2-deoxy-D-glucan (Adamas-beta, Shanghai, China) to the liposome suspension. The solutions were stirred at 37 °C for 1 h and then strained 20 times through a 100 nm filter at 58 °C. The free drugs were removed by centrifugation through an Amico filter device (MWCO = 10 kDa) and washed with PBS four times.

Characterization of the prepared DDS

The diameter and zeta potential of the liposomes were measured by dynamic laser scattering (DLS, DynaPro Plate Reader II, Wyatt) after dilution with sterile 1 × PBS. For visual characterization of the DDS, the liposomes were loaded onto formvar-coated nickel grids (Electron Microscopy Sciences) and negatively stained using 2% phosphotungstic acid solution (Sigma). The morphology of the liposomes was then imaged at 120 kV on an HT7700 TEM (Hitachi) in conbinaiton with a digital micrograph camera and software suite (Gatan).

Testing the drug loading capacity of DDS

Since the mixing ratio of drug to liposomal material was 1:1, we tested the drug loading as represented by metformin hydrochloride. The DDS was treated with Triton X-100 to disintegrate the phospholipid bilayers and release the encapsulated drugs. High Performance Liquid Chromatography (HPLC) was then applied to determine the concentration of the released metformin hydrochloride. To test the concentration of metformin hydrochloride in the DDS, metformin hydrochloride (0.165 mg in 1 mL solution) was utilized as the internal standard solution, while metformin hydrochloride solutions with different concentrations were utilized as the reference. The broken liposomes were purified for HPLC analysis.

***In vitro* experiments**

Cellular proliferation assays were performed using the CCK8 Kit (Beyotime Biotechnology) in accordance with instructions provided by the manufacturer. For cell viability measurement, cells were cultured into 96-well plates at least triplicate and then subjected to drug treatment as indicated. Western blot was used to detect the proteins after electrophoresis. p-eIF2α Rabbit pAb (Catalog No. R22946), eIF2α Rabbit mAb (Catalog No. 310073), ATF4 Rabbit mAb (Catalog No. 381426) and β-Tubulin mouse mAb (Catalog No. A12289) were purchased from zenbio Technology Co., Ltd (chengdu, China). Annexin V-FITC Apoptosis Detection Kit (Beyotime Biotechnology) was used for detection of apoptosis. The lactic acid concentration in cell culture medium was detected by Lactic Acid assay kit (Nanjing Jiancheng Bioengineering Institute, Nanjing, China).

***In vitro* transwell migration and invasion**

Cell migration and invasion experiments were performed using 24-well plates with 8 μm-polycarbonate filter inserts (#3422, Corning). CT26 cells were seeded at densities of 2×10^5 cells/200 μL and 1×10^5 cells/200 μL per well, respectively, in serum-free RPMI 1640. All cells were either uncoated or Matrigel-coated and incubated in chambers containing 600 μL of RPMI 1640 with 10% foetal serum as a chemoattractant. The cells were imaged, and their migration and invasion were captured using a microscope (Nikon, Eclipse Ti-S). All experiments were performed thrice independently.

Plate colony formation assay

CT26 cells with different treatments were independently seeded in six-well plates at densities of 5000 cells per well and incubated at 37 °C. The medium was RPMI 1640 containing 10% foetal bovine serum, which was changed every other day. After 10 days, colony-forming cells were immersed in 4% paraformaldehyde

for 20–30 min, stained with crystal violet for 2 h, and then rinsed three times with PBS to remove the excess crystal violet. Finally, images were captured using a microscope, and the number of colony-forming units was counted.

Cell apoptosis analysis

To analyze the fraction of apoptotic cells, CT26 cells with different treatments were evaluated using the annexin V-APC/7-AAD apoptosis detection kit (KA3808, Abnoya). Briefly, each sample containing 1×10^5 cells was washed twice with cold phosphate-buffered saline (PBS) and then suspended cells in 100 μL 1 × binding buffer. Then, 5 μL of 7-AAD and 5 μL of APC annexin V were added to each sample. The cells were incubated in the dark for 15 min at room temperature. Approximately 10,000 cells/sample were analyzed by flow cytometry (Becton, Dickinson and Company, FACS Canto II).

γH2AX immunofluorescence assay

γH2AX, as a critical marker of DNA double-strand breaks, was assessed in CT26 cells. In this process, cells were first cultured in a 6-well plate with a seeding density of 1×10^6 cells per well. After 24 h, the cells underwent a washing step with PBS and were subsequently exposed to 8Gy of irradiation. Following an additional 12 h of incubation, the cells were immunostained using a specific γH2AX antibody. The confocal images of cells were performed using an Olympus FV1200 microscope to analyze the formation and distribution of γH2AX foci in the cells.

Tumor model

CT26 cells cancer cells (2×10^6) in 50 μL PBS were injected into the back of BALB/c mouse to establish the subcutaneous tumor model. BALB/c mouse were intravenously injected with Luciferase-CT26 cells (2×10^5 cells per mouse) suspended in 0.2 μL of PBS to establish the CRC in situ tumors model. All mice were randomly divided into groups for treatment experiments. The tumor size was calculated as: length × width^2 × 0.5. The tumor-bearing mice were euthanized and sacrificed once the volume of tumor reached 1000 mm^3.

***In vivo* biodistribution**

The biodistribution of liposome nanoparticles in mice was monitored using an IVIS imaging system (PerkinElmer) at 2, 8, 12, 24 and 48 h after systemic administration ([DIR-labeled DDS] = 100 μg/mice). The tumors and tissues were collected from mice 48 h after i.v. injection and then placed on solid black paper for fluorescence imaging. The fluorescence intensity was measured using an IVIS imaging system (PerkinElmer) and quantified using Living Image software (PerkinElmer). The bilateral CT26 tumor-bearing mice were sacrificed 48 h after systemic administration of DIR-labeled M/D-Lipo to analyze their biodistribution behavior. The fluorescence images of mice, organs and tumors were photographed by IVIS.

Combination therapy and related mechanisms

The treatment plan was conducted when the average tumor size reached 50 mm^3. Liposomes (M/D-Lipo ([M] = 2 mg/mouse) were intravenously injected into mice. Mice were anesthetized by intraperitoneal injection of

4% chloral hydrate (400 mg/kg) and then mechanically immobilized in a fixture for X-ray irradiation (radiation dose rate: 113 cGy/min, RS-2000 Pro irradiator), which exposed the tumor or lower abdomen to the radiation field while shielding the rest of the body from radiation exposure. Ten days after local tumor treatment, mice were sacrificed to collect the tumors for immunofluorescence analysis of PD-L1. Five days after the treatments, the immune cells in the distant tumors and lymph nodes were analyzed by immunofluorescence slices and flow cytometry after staining with antibodies. After one week of different treatments, tumors were collected and stained with H&E for histopathological analysis.

Statistical analysis

The data are expressed as the mean plus standard deviation unless otherwise stated. T tests were used for comparisons. All statistical analyses were performed using Excel 2016 and GraphPad Prism 8 for Windows (GraphPad Software). The threshold for statistical significance was $P < 0.05$.

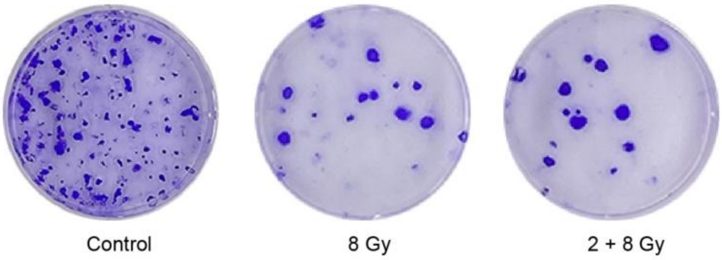

Figure S1. The clonogenic ability of CT26 cells after conventional fractionated radiotherapy was stronger than that after single fraction radiotherapy.

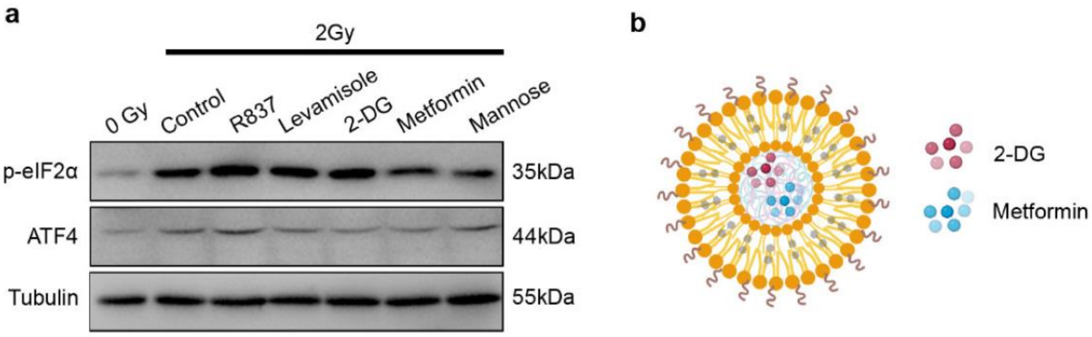

Figure S2. (a) Western blot characterized p-eIF2α and ATF4 expression in CT26 cells after different treatments (Control, 2Gy, 2Gy+R837 (0.1 mg/ml), 2Gy+Levamisole (0.1 mg/ml), 2Gy+2-DG (0.1 mg/ml), 2Gy+Metformin (0.1 mg/ml), 2Gy+Mannose (0.1 mg/ml). (b) Schematic representation of the synthesis of drug-loaded liposomes.

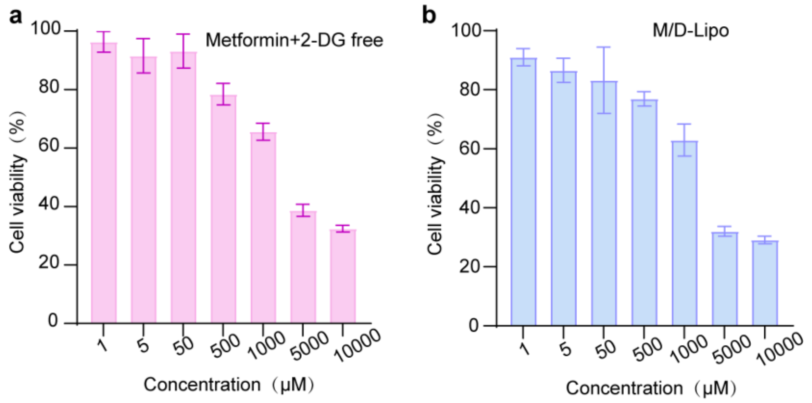

Figure S3. (a) The relative cell viability of CT26 cells incubated with free metformin and free 2-DG at different concentrations for 24 h. Error bars represent mean ± s.d. (n = 6). (b) The relative cell viability of CT26 cells incubated with M/D-Lipo at different concentrations for 24 h. Error bars represent mean ± s.d. (n = 6).

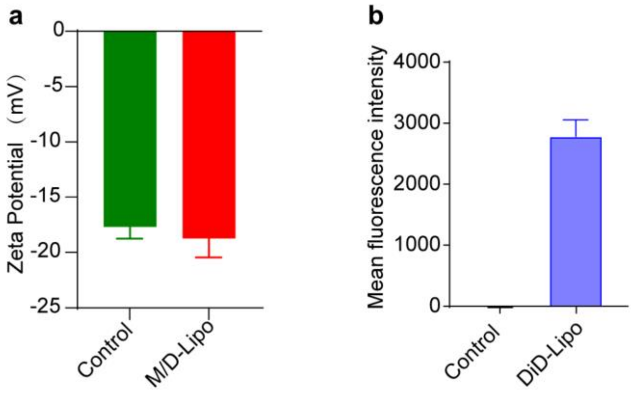

Figure S4. (a) Zeta potential of Lipo, M/D-Lipo, measured by DLS. Error bars represent mean ± s.d. (n = 3). (b) The fluorescence content of DiD-labeled liposomes was determined by flow cytometry compared with that of DiD-unlabeled liposomes. Error bars represent mean ± s.d. (n = 3).

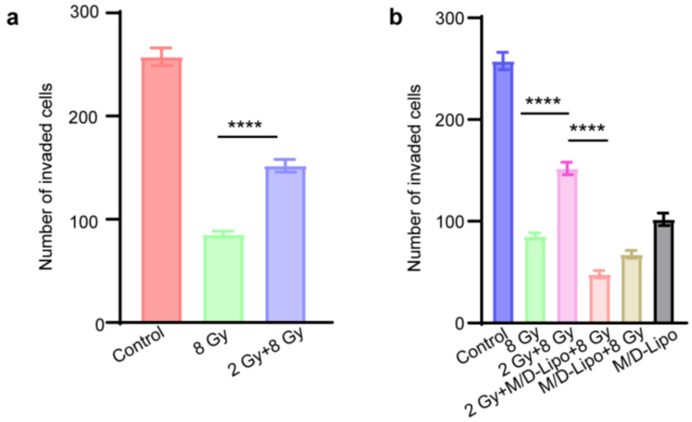

Figure S5. (a) The number of invasive cells was counted in the cell invasion assay. (Control, 8 Gy, 2 Gy+8 Gy). Error bars represent mean±s.d. (n = 6). (b) The number of invasive cells was counted in the cell invasion assay. (Control, 8 Gy, 2 Gy+8 Gy, 2 Gy+M/D-Lipo+8 Gy, M/D-Lipo+8 Gy, M/D-Lipo)Error bars represent mean±s.d. (n = 6). *P* values were calculated by Student's t tests (*****P* < 0.0001).

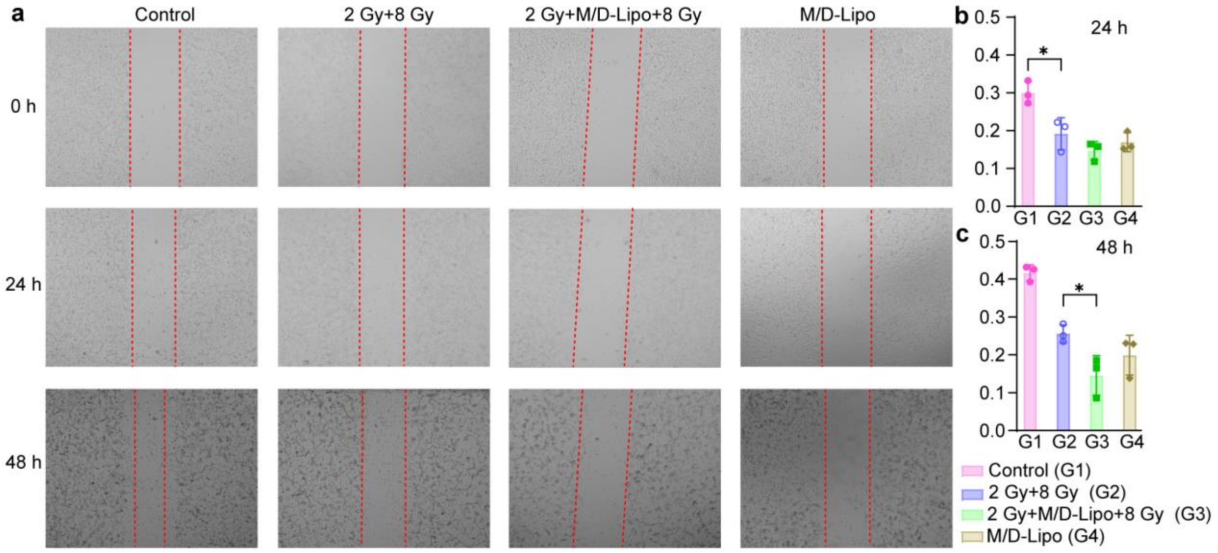

Figure S6. (a) Cell scratch assay verified the cell migration rate(Control, 2 Gy+8 Gy, 2 Gy+M/D-Lipo+8 Gy, M/D-Lipo). (b-c) Cell spacing was measured at 24h (b) and 48h (c) after treatment.Error bars represent mean±s.d. (n = 3). *P* values in b and c were calculated by Student's t tests(**P* < 0.05)

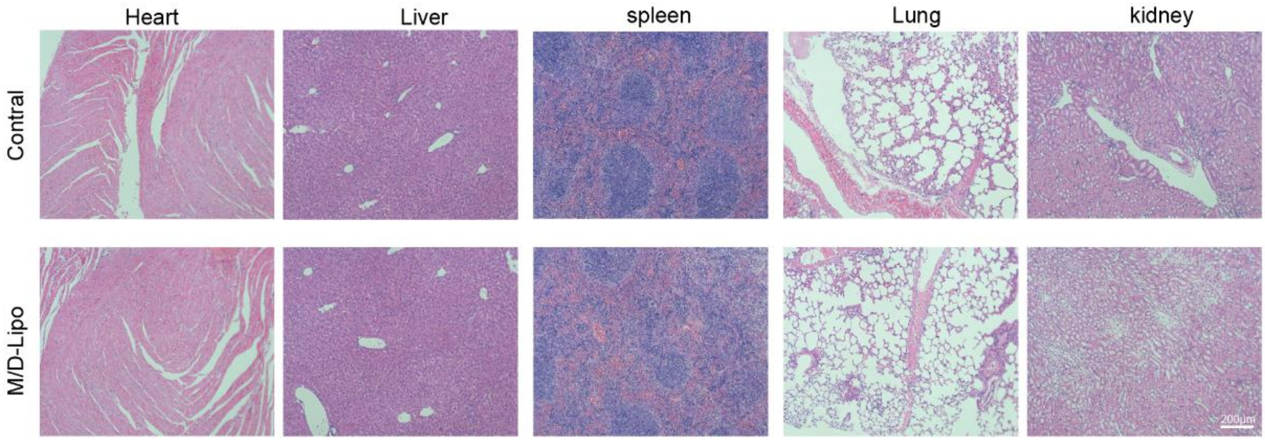

Figure S7. Micrographs of H&E-stained tumor slices at Day 7 (Control, M/D-Lipo) (scale bar:200 μm).

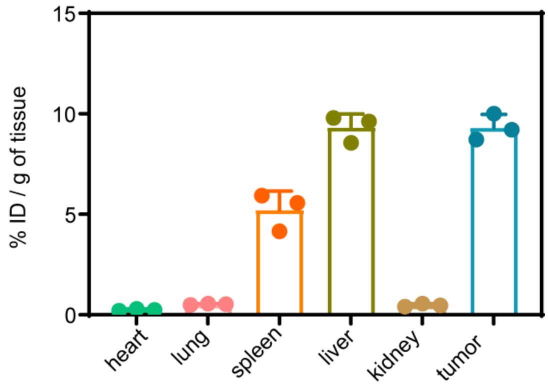

Figure S8. The 48 h organs accumulation values (mean ± SD) of DIR-labeled M/D-Lipo. The 48 h organs accumulation values (mean ± SD) of DIR-labeled M/D-Lipo were, 0.25 ± 0.04 %ID/g (heart); 0.53 ± 0.03 %ID/g (lung); 5.22 ± 0.94 %ID/g (spleen); 9.32 ± 0.67 %ID/g (liver); 0.476 ± 0.075 %ID/g (kindney); 9.31 ± 0.66 %ID/g (tumor).

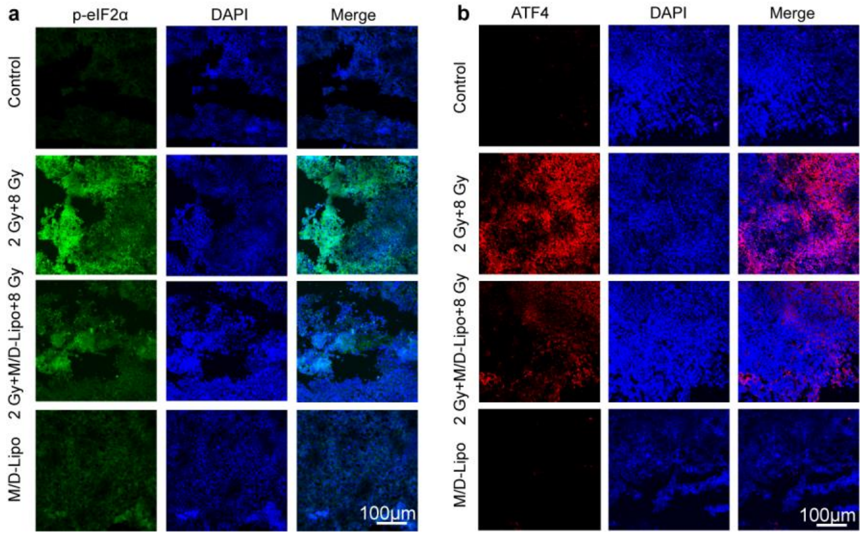

Figure S9. (a) Immunofluorescence staining showed p-eIF2α protein (green) in the subcutaneous tumor after 5 day of different treatments (scale bar:100 μm). (b) Immunofluorescence staining showed ATF4 protein (rad) in the subcutaneous tumor after 5 day of different treatments (scale bar:100 μm).

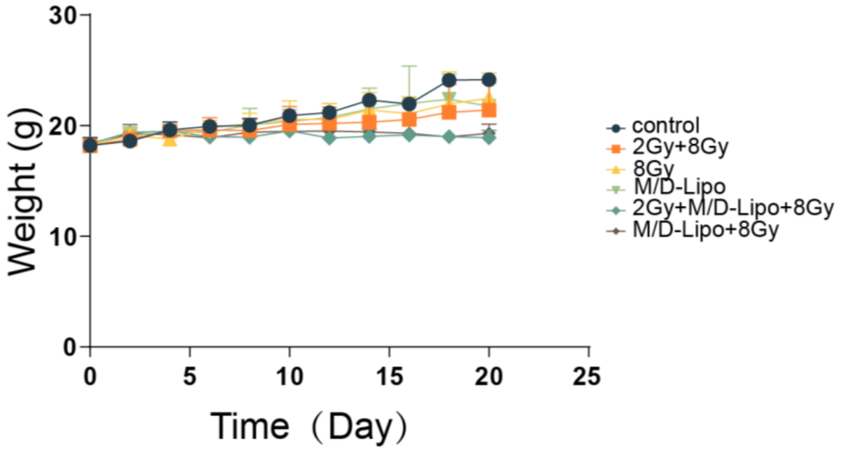

Figure S10. The weight growth curves of mice with single tumor after different treatments (Control, 2 Gy+8 Gy, 8 Gy, M/D-Lipo, 2 Gy+M/D-Lipo+8 Gy, M/D-Lipo+8 Gy) (n = 5).

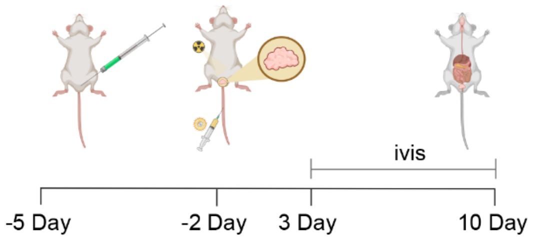

Figure S11. Schematic of orthotopic mouse colorectal construction and treatment.

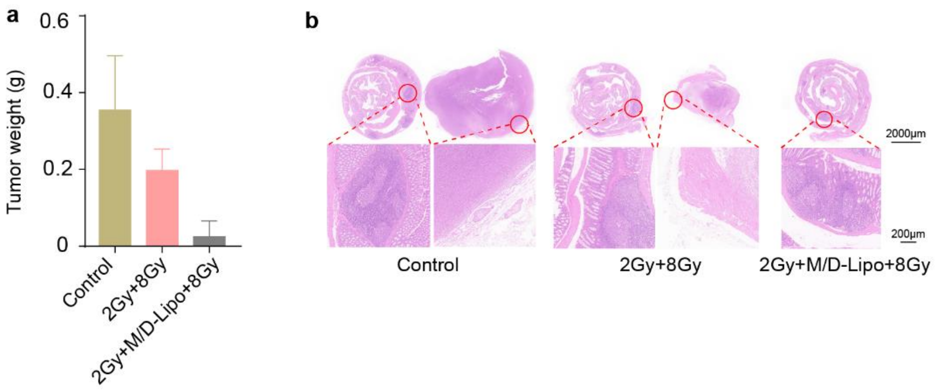

Figure S12. (a) Weight statistics of orthotopic colorectal tumors in mice after different treatments (Control, 2Gy+8Gy, 2Gy+M/D-Lipo+8Gy). Error bars represent mean±s.d. (n = 5). (b) Micrographs of H&E-stained tumor slices at Day 7 (Control, 2 Gy+8 Gy, 2 Gy+M/D-Lipo+8 Gy) (scale bar:200 μm).

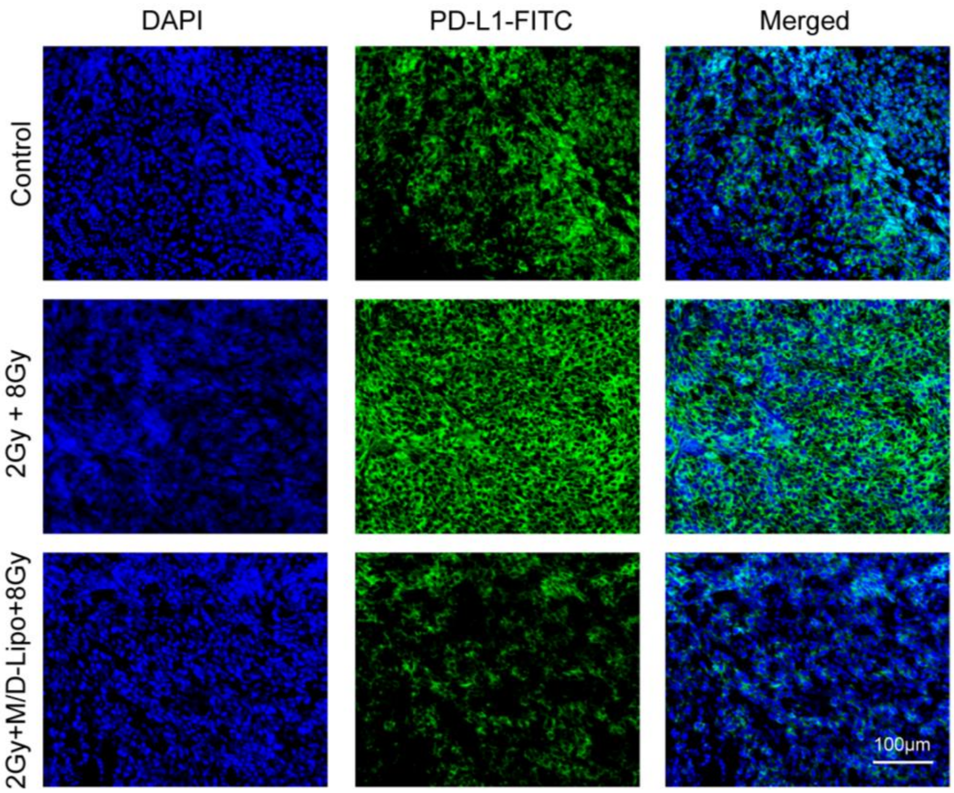

Figure S13. Immunofluorescence images of PD-L1 in CT26 cells at 24h post different treatments (Control, 2 Gy+8 Gy, 2 Gy+M/D-Lipo+8 Gy) (scale bar 100 μm).

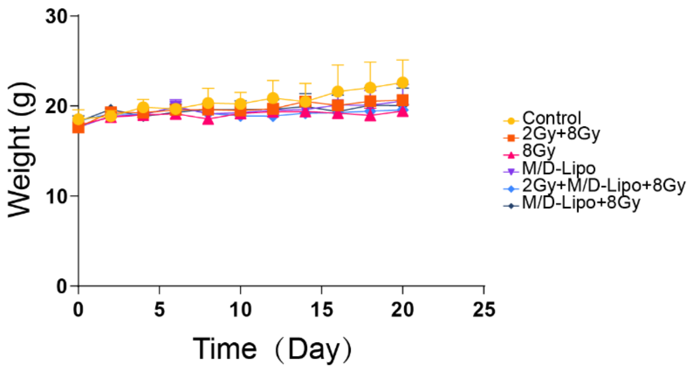

Figure S14. The weight growth curves of mice with metastatic tumor after different treatments (Control, 2 Gy+8 Gy, 8 Gy, M/D-Lipo, 2 Gy+M/D-Lipo+8 Gy, M/D-Lipo+8 Gy) (n = 5).
